# Supplementary material for: Are carbon emissions trading and green financial instruments synergistic? -Comprehensive quantitative research based on content analysis
Source: PLoS One. 2024 Mar 7;19(3):e0298601. doi: 10.1371/journal.pone.0298601 (PMC10919601; doi:10.1371/journal.pone.0298601)
Supplement: S1 Data — (ZIP) [file pone.0298601.s001.zip › Data/地级市Z_F工作报告.zip!/┴╔─■/╔≥╤⌠╩╨/└┤╫╘╣1⁄2╓┌║┼í╛┬φ┐╦╩2╛▌═°í┐_2015.docx]

各位代表：

　　现在，我代表市政府向大会报告工作，请予审议，请市政协各位委员提出意见和建议。

　　一、2014年工作回顾

　　过去一年,面对严峻的经济形势和艰巨的改革发展任务,在市委的正确领导下，在市人大及其常委会依法监督和市政协民主监督下，市政府紧紧依靠全市人民，深入贯彻落实党的十八大和十八届三中、四中全会精神，抢抓中央实施新一轮东北振兴战略的重大机遇，扎实推进“深化改革、稳定增长、改善民生、优化环境”等重点工作，全市经济社会实现了持续稳定发展。

　　——经济运行总体平稳。预计全市地区生产总值实现7589亿元，比上年增长6%；一般公共预算收入完成785.5亿元，下降1.9%；固定资产投资完成6575亿元，增长3%；社会消费品零售总额实现3568.4亿元，增长12%。

　　——重点领域改革扎实推进。行政体制改革步伐加快，市政府工作部门由45个精简到40个，取消和下放行政职权649项，国有企业、非公经济、金融体制等各项改革取得积极进展。

　　——城乡环境持续改善。重点基础设施建设项目和城乡环境改造项目有序推进，城市精细化管理水平进一步提升。全市单位地区生产总值能耗下降4.5%,化学需氧量、二氧化硫排放总量分别下降2.5%和1.3%。

　　——人民生活水平不断提高。城市居民人均可支配收入实现31720元，增长9.1%；农村居民人均可支配收入实现15945元，增长10.2%。

　　——社会更加和谐稳定。社会事业蓬勃发展，社会保障水平不断提升，社会治理体系逐步完善，安全生产形势保持总体平稳，“平安沈阳”建设取得明显成效。

　　一年来，我们主要抓了以下几方面工作。

　　(一)全力以赴稳增长，经济保持平稳健康发展

　　加强经济运行组织协调。制定实施促进经济稳增长若干政策措施，开展涉企收费清理整顿工作，取消、缓征11项行政事业性收费和政府性基金项目，减轻了企业负担。深入开展“双进双解”活动，加强生产要素供给，帮助企业特别是中小微企业解决困难和问题，有力促进了实体经济健康发展。认真落实国务院28号文件精神，重大政策和项目争取落实工作取得积极进展。

　　注重发挥有效投资的关键作用。坚持以项目带动投资、以投资促进发展,开展社会资本投资交通、环保、社会事业等12个领域试点，加快基础设施和重大产业项目建设。116个政府投资项目完成投资105亿元，开复工投资亿元以上项目1535个，完成投资5144.5亿元，其中290个重点项目完成投资2153.9亿元。

　　千方百计促进出口、拉动消费。出台促进外贸稳定增长、提高贸易便利化水平等政策措施,全市进出口总额158亿美元，增长10.6%。支持开展节庆促销，大力发展夜间经济，积极培育信息、文化、旅游等新的消费热点，有效激活了大众消费。

　　(二)加大结构调整力度，经济发展质量效益稳步提高

　　工业经济实力不断增强。全市规模以上工业增加值实现3700亿元，增长5.8%，其中规模以上装备制造业增加值1813亿元，增长8%。以建设先进装备制造业基地为目标，积极促进传统产业升级，大力培育新兴产业，以智能制造为重点的两化融合取得新突破，我市成为全国唯一的建筑产业现代化示范城市。汽车产业拉动作用明显，全市整车产量108.7万辆，汽车及零部件制造业产值2218.1亿元，增长13.9%。智能机器人、航空、电子信息等战略性新兴产业加速发展，沈阳国家级互联网骨干直联点开通，东北区域超算中心、北斗(辽宁)卫星运营信息服务平台投入使用，中航工业波音制造创新中心落户浑南，法库通用航空产业基地建设扎实推进。沈鼓集团、沈飞集团获中国工业大奖，新松公司入选中国市场最具竞争优势移动机器人企业十强。全市高新技术产品增加值实现1740亿元，增长9.6%。

　　服务业加快发展。全市服务业增加值实现3371.1亿元，增长6.2%；现代服务业占服务业比重达到54%。启动实施服务业发展四年行动计划，物流、会展、文化、旅游等产业稳步发展，金廊核心段项目加快建设，工业企业分立出生产性服务业企业282户。全市新增金融及金融服务机构54家，盛京银行、禾丰牧业、萃华珠宝实现上市，帝信通信、中科仪等11户企业在“新三板”挂牌。我市获批创建国家电子商务示范城市。

　　农业和县域经济全面发展。持续加强农田水利等基础设施建设，采取有效措施科学抗旱，粮食产量达到72亿斤，实现“十一连丰”。蒲河生态经济带、沈康现代农业示范带、环城都市农业产业带建设扎实推进，全市建设粮油作物高产示范区119个，新建规模以上农业产业化项目80个，重点龙头企业达到266家。县域工业稳步发展，金杯卡车搬迁改造项目加快推进，玖龙纸业试生产，11个产业集群实现销售收入1690亿元。县城和中心镇建设步伐加快，新型城镇化取得新进展。

　　(三)全面推进改革开放，经济社会发展活力不断增强

　　十方面改革不断深化。行政体制改革率先突破，政府机构改革加快推进，开发区管理机构由31个减少到13个，注销、撤销各类协会75个，简政放权工作走在全省前列。国有企业改革稳步推进，东药集团完成定向增发，沈鼓集团完成私募股改和员工持股。非公经济改革取得成效，登记制度改革深入推进，率先开展企业“三证合一”试点，全市新登记各类企业2.3万户，增长74.5%。统筹城乡区域改革加快实施，农村土地产权制度改革步伐加快，土地流转103.9万亩，确权登记颁证工作全省领先，新民市获批省级农业现代化与新型城镇化综改试验区。金融体制改革扎实推进，积极发展民营银行、金融租赁公司等新型金融机构，辽宁股权交易中心设立国内首个区域科技股权交易板块。科技体制改革取得新成果，组建IC装备、机器人、硬岩掘进机等32个产业技术创新战略联盟，远大科技园经验在全国推广，全市有效发明专利占全省40%。生态文明体制改革取得新突破，完成第一批生态保护红线区划定工作。社会治理体制创新稳步实施，铁西区、沈河区成为全国社会组织创新管理示范区。

　　对外开放取得积极进展。积极参与央企、沪企、民企辽宁行活动，一批重大项目落户沈阳。中法沈阳生态园经贸合作协议成功签署，中德装备园启动建设。企业“走出去”步伐加快，境外协议投资额3.7亿美元，增长86.6%。成功举办“第十四届中国制博会”、“中国(沈阳)国际金融高层峰会”、“第六届中欧投资贸易洽谈会”、“沈阳法库国际飞行大会”等大型经贸活动，进一步提升了沈阳的知名度和影响力。沈阳经济区建设步伐加快。交通、旅游、通讯等重点领域一体化取得新突破，沈抚同城化取得新进展，本溪成为024电信区号共享城市，城际连接带新城新市镇建设全面加快。

　　(四)加强城乡建设管理，城市功能环境进一步优化

　　基础设施建设迈上新台阶。全年城建投资完成308亿元。二环快速路二期、沈阳北站北广场改造、迎宾路高架桥、东陵路高架桥、浑河云龙湖桥等项目开工建设，地铁9号线和10号线、沈铁城际铁路(沈阳段)、沈阳南站等项目建设加快，谟家堡跨三环桥、东望街延长线建成通车，长客西站、长客总站综合交通枢纽投入使用。实施市区老旧泵站、积水点改造，完成131公里供水管网、107公里供热管网改造及4处热网互联工程，城市运行保障能力进一步增强。

　　生态环境持续改善。拆除20吨以下锅炉房134座，完成新北热电等8家电力企业脱硫、脱硝改造和95台燃煤锅炉清洁能源改造。加强辽河、浑河、蒲河等重点流域治理，开展村镇生活污水处理设施运行专项整顿，3座新建污水处理厂投入运行。开展超坡地还林、荒山造林和封山育林，推进三四环沿线生态绿带建设，全市植树造林42.8万亩。积极推进城乡垃圾减量化、无害化、资源化处理，老虎冲垃圾场渗沥液处理设施改扩建等项目加快推进。涉农区县(市)全部成为国家生态文明建设示范区。

　　城市管理水平进一步提升。积极构建市民公共服务体系，加大市政设施养护力度，沈河区“先予处置”城市维护管理模式在全市推广。加快立体公共停车场、行人过街设施建设，建成智能交通指挥平台，新增更新公交车130辆，新开调整公交线路26条。开展建筑工地、道路卫生保洁等重点领域专项整治，加强农村环境连片整治和宜居乡村建设，城乡人居环境进一步改善。

　　(五)着力保障改善民生，基本公共服务水平持续提升

　　民生工作扎实推进。加强对就业、创业的援助和扶持，困难家庭高校毕业生全部就业，零就业家庭保持动态为零，城镇登记失业率为3.03%。完成102.5万企业退休人员养老金提标发放工作，城镇居民医保和新农合年人均补助由280元提高到320元，城乡低保救助标准分别提高10.2%和10.5%，医疗救助标准提高10%。新建社区老年人日间照料站50个，新增养老机构床位4760张，我市成为全国养老服务业综合改革试点城市。残疾人就业、康复、教育等工作有效推进。完成3.1万套保障性住房建设、1.2万户棚户区和1433户农村危房改造任务，完成100个旧住宅区环境改造和40个旧住宅区节能暖房改造，建成80个公交站务室和144处环卫工人休息室，解决了18.6万农村人口饮水安全问题，向市民承诺的12件33项民生实事全部完成。

　　社会事业全面发展。完成100所幼儿园改造，新增普惠性幼儿园学位1.5万个,建设300所中小学数字校园，完成110所义务教育学校、34所普通高中标准化建设，大东、于洪、苏家屯等5个区(县)被评为国家义务教育发展基本均衡县。辽中、法库县级公立医院改革全面启动，基层医疗卫生机构信息化建设稳步推进，市胸科医院一期、四院新门诊楼投入使用。全面实施文化基础设施提升工程，盛京大剧院投入使用，市青少年活动中心、妇女会馆、少儿图书馆、老年人大学等新建、改建项目开工，市全民健身中心和15处市民休闲广场建成开放，群众文化体育生活更加丰富多彩。

　　社会治理不断加强。强化安全生产责任制，推进安全生产制度化、标准化、网格化、信息化建设，安全生产形势持续稳定。调整管理体制，加强食品药品安全监管，严厉打击违法行为。加强治安防控管理体系建设，有针对性地实施打击整治专项行动，开展“零发案”小区(村屯)三年创建活动，人民群众安全感不断增强。深入开展矛盾纠纷排查，积极化解信访事项，依法保障人民群众合法权益，我市被评为全国和谐社区建设示范城。全国文明城市创建工作深入推进，城市文明程度和市民文明素质实现新提升。

　　过去一年，我们坚持依法行政，加强行政决策合法性审查，进一步规范行政自由裁量权，巩固群众路线教育实践活动成果，开展“转作风、转职能、优环境”专项整治，政府自身建设取得新成效。国防动员、人防和民兵预备役建设进一步加强，双拥共建工作全面推进。民族团结进步事业不断发展，对口支援工作成效显著。国家安全、保密、统计、审计、参事、宗教、侨务、贸促、档案、修志、文史、红十字会和慈善事业等各项工作都取得了新成绩。

　　各位代表，过去一年所取得的成绩来之不易，这是市委正确领导的结果，是全市人民凝心聚力、开拓奋进的结果。在此，我代表市政府，向全市各族人民致以崇高的敬意！向人大代表、政协委员，向各民主党派、工商联、无党派人士和各人民团体，向驻沈人民解放军、武警部队官兵和中、省直单位，向所有关心、支持沈阳的港澳台同胞、海外侨胞和国际友人表示最衷心的感谢！

　　在总结成绩的同时，我们也清醒地认识到存在的问题。一是尽管去年我市主要经济指标增速都高于全省平均水平，但地区生产总值、一般公共预算收入、固定资产投资、社会消费品零售总额等经济指标未能完成年初人代会确定的预期目标。二是转方式、调结构的成效还未充分显现，传统产业优势有所减弱，新兴产业规模不大、实力不强，自主创新能力亟待提升，支撑经济平稳增长的动力不够强劲。三是人民群众关心关注的一些热点难点问题还没有完全解决到位，保障改善民生任务依然繁重。四是政府职能和工作作风需要进一步转变，服务效率需要进一步提高。

　　分析部分指标未完成的原因：一是受宏观经济大环境影响，国内经济进入“三期叠加”阶段，老工业基地尚未根本解决的一些体制性、机制性、结构性矛盾更加凸显。二是落实对企业减免税费、扩大“营改增”等政策措施，相应减少了财税收入。三是我们认真落实国家和省关于经济指标的各项要求，坚持实事求是原则，使经济指标更有质量、更加真实。四是针对经济运行中出现的困难和问题，我们虽然主动采取了应对措施，但受政策效应滞后等因素影响，效果还没有充分显现。

　　对工作中存在的问题，我们将高度重视，采取有力措施，认真加以解决。

　　二、2015年工作安排

　　今年是全面深化改革、全面推进依法治国、全面贯彻落实中央新一轮东北振兴战略的关键一年，也是全面完成“十二五”规划的收官之年，做好今年各项工作意义重大。当前，世界经济总体复苏疲软态势没有明显改观，我国已经进入经济发展新常态，我市经济结构调整、产业转型升级、新经济增长点培育、稳增长任务仍然十分艰巨。我们要紧紧抓住中央加快实施新一轮东北振兴、“一带一路”、京津冀协同发展等重大战略机遇，积极适应新常态，开创发展新局面。

　　今年政府工作的总体要求是：全面贯彻党的十八大和十八届三中、四中全会及中央经济工作会议精神，认真落实习近平总书记系列重要讲话和重要批示精神，按照省委省政府、市委的要求和部署，坚持稳中求进工作总基调，坚持以提高经济发展质量和效益为中心，坚持以转方式调结构为主线，突出抓好改革创新、稳定增长、转型升级、改善民生、依法行政等重点工作，促进经济持续健康发展、社会和谐稳定，推动沈阳老工业基地在新一轮东北振兴中当先锋、打头阵。

　　今年全市经济社会发展的主要预期目标是：地区生产总值增长6%，固定资产投资增长5%，一般公共预算收入增长2.5%，社会消费品零售总额增长11%，城市居民人均可支配收入增长7%，农村居民人均可支配收入增长8%，城镇登记失业率控制在4%以内。

　　今年要重点抓好以下几方面工作。

　　(一)全面贯彻中央支持东北振兴战略

　　贯彻落实中央新一轮东北振兴战略是当前政府工作的重中之重，必须抓紧抓好，抓出成效。

　　认真落实各项支持政策。按照中央提出的财税、金融、投资、产业、创新等领域重点支持政策，主动配合、抓紧对接，努力在提升产业竞争力、深化国企改革、加快重大基础设施建设、加强生态保护、扩大开放合作、保障改善民生等各个方面实现新突破，切实推动经济发展提质增效升级。

　　抓紧推进重点工作任务。全力以赴落实国务院28号文件明确指向沈阳的政策和项目，加快推进沈阳中德装备园、沈阳军民融合发展示范区建设等重点任务，力争政策效益最大化。推动本地企业与央企、沪企、知名民企开展合作，促进企业转型升级、做大做强。

　　积极开展先行先试。按照国务院提出的试点方向，大胆进行改革试验，为争取更多的国家试点创造有利条件。努力争取设立东北振兴基金、发展民营银行等金融改革试点，大力发展产业金融，为破解中小微企业融资难问题、加快产业转型升级提供有力支撑。

　　(二)保持经济稳定增长

　　把稳增长作为经济工作的第一要务，更加注重经济增长的质量和效益，保持经济运行处于合理区间。

　　着力抓好有效投资。充分发挥政府投资拉动作用，加快提升基础设施和民生服务水平。鼓励社会资本投资社会事业、民生等项目，进一步释放民间投资潜力。优化投资结构，加强组织调度，推进270个重点项目加快开工建设、早日达产达效。加强项目储备，不断增强发展后劲。

　　努力扩大消费需求。通过增加城乡居民收入，提高消费能力，进一步活跃大众日常消费。积极改善消费环境，培育新的消费热点，重点推进信息、住房、旅游休闲、教育文体、养老健康等领域消费，充分释放全社会巨大的消费潜力。

　　优化企业发展环境。落实支持实体经济的各项政策措施，着力减轻企业负担。加强软环境建设，继续开展“双进双解”活动，做好各类生产要素保障供应，及时帮助企业解决发展中遇到的困难和问题。更加重视和关心中小微企业发展，着力解决企业在融资、创新、成长等方面存在的实际困难，让中小微企业享受到更多的阳光雨露，不断为经济发展注入新能量。

　　(三)全面深化改革扩大开放

　　改革开放是破解体制机制障碍、加快沈阳老工业基地振兴的根本之策，必须毫不动摇地全面加以推进。

　　深入推进重点领域改革。认真落实市委改革领导小组的决策部署，全面深化新型工业化综合配套改革，突出抓好十方面重点领域改革。推动东药集团、中兴商业集团等国有企业不断完善现代企业制度，推进北方重工、机床集团等国有企业与央企战略重组，加快沈鼓集团上市步伐,积极推进国有公交企业改革。鼓励非公有制企业参与国有企业改革，积极发展混合所有制经济。加快城乡统筹发展步伐，扎实做好于洪、苏家屯、新民、法库等省级改革试点工作。深入推进农村产权制度改革，稳步开展农村集体土地及资产确权登记颁证工作，积极推进土地流转，鼓励发展多种形式适度规模经营和集约经营。稳妥推进事业单位分类改革。扎实做好公务用车制度改革。按照简政放权、放管结合新要求，深入推进行政体制改革，加快建立行政权力清单和涉企收费清单制度，积极营造全民创业、全社会创新的良好环境，充分激发市场和社会发展活力。

　　进一步扩大对内对外开放。加大重点产业招商力度，吸引更多世界500强、行业领军企业投资落户沈阳，着力引进具有牵动力的重大项目，不断提升招商引资的质量和效益。培育保税物流、跨境电子商务、软件服务外包等新兴业态，推进出口基地建设，提升沈阳综合保税区功能，深化关检“三个一”合作，努力扩大外贸出口。实施企业“走出去”战略，支持优势企业开展境外投资、建立生产基地、承包国际工程，增强国际化经营能力。继续以沈抚同城化为突破口，推进城际连接带新城新市镇建设，加快主导产业园区发展，促进资源共享、产业集聚和各类要素市场一体化发展。

　　(四)大力实施创新驱动

　　创新是培育新的增长动力和形成新的竞争优势的关键，必须充分发挥科技和人才优势，加快国家创新型城市建设步伐。

　　加快构建技术创新体系。坚持需求导向和产业化方向，深化院市合作、校企合作，推动沈阳材料国家实验室、中科院机器人与智能制造创新研究院等新型研发实体建设。进一步强化企业创新主体地位，加快产业技术创新战略联盟建设，鼓励自主创新，推进协同创新。努力争创国家自主创新示范区。

　　积极推进科技成果转化。加大研发投入，深入实施重大科技专项，进一步提升核心技术研发能力。大力推广本地企业首台(套)重大技术装备，鼓励本地技术装备在国家重点工程中的应用。加大知识产权创造、运用、保护和管理力度，健全技术创新激励机制，支持企业建立科技研究院，促进科技成果资本化、产业化。

　　加强人才队伍建设。进一步完善人才政策，努力营造留住人才、吸引人才、聚集人才、用好人才的良好环境，加大领军人才和研发团队的培养、引进力度，为实现创新驱动发展提供强有力的智力支撑。

　　(五)加快产业优化升级

　　坚持走新型工业化道路，加快产业结构调整步伐，努力构建结构合理、优势突出、竞争力强的现代产业体系。

　　推进工业转型升级。坚持创新驱动，促进两化深度融合，推动装备制造业不断提高自主创新能力、重大装备成套能力、基础产业配套能力和生产性服务业支撑能力，努力打造装备制造业升级版。以龙头企业为重点，着力提升汽车及零部件、机械装备、现代建筑、农产品加工等优势产业整体发展水平和市场竞争力。优化产业布局，加快重大项目、重点园区和产业集群建设，推进产业集聚发展。大力发展高端装备制造业，培育壮大机器人、燃气轮机、IC装备及零部件等重点产业，加快新松产业园建设，打造世界级机器人产业基地。加强云计算、物联网、大数据等新技术的开发应用，加快卫星导航与位置服务、软件信息、移动互联网等产业发展。大力发展通用航空产业，重点支持沈飞民机、西子航空、联航神燕等龙头企业发展，规划建设空港经济区，做大做强临空产业。全面发展生物医药产业，积极支持药物新产品、医学装备研发和产业化。

　　加快发展现代服务业。继续实施服务业发展四年行动计划，支持铁西区创建国家生产性服务业集聚发展示范区，推进金廊等现代服务业集聚区优化升级，加快太原街、中街等传统商业街区业态提升。积极引进金融机构区域性总部，加强沈阳金融商贸开发区、和平金融街和皇姑北站北等金融集聚区建设。积极引进电商平台和商务楼宇品牌运营商，加快传统批发、零售业转型步伐，鼓励支持企业发展和应用电子商务，加速形成现代物流产业体系。加快国际会展商贸城建设，积极申办国内外大型展会，努力打造中国北方商务会展中心城市。深入挖掘文化旅游、健康休闲资源，优化景区景点布局，加快发展文化旅游和体育休闲产业。积极应对、综合施策，促进房地产业平稳健康发展。

　　积极发展现代农业。认真落实中央一号文件精神，按照稳粮增收、提质增效、创新驱动的总要求，兑现各项强农惠农富农政策。加强农田水利等基础设施建设，保障粮食和主要农产品有效供给。以蒲河生态经济带、沈康现代农业示范带和环城都市农业产业带为载体，促进高端精品、高效特色、高产生态、休闲观光等都市现代农业发展。培育家庭农场、休闲农庄等新型农业经营主体，大力发展农民专业合作社和农业产业化龙头企业，强化农业科技服务，不断完善农业社会化服务和产业化经营体系。加强“新网工程”建设，加快发展农产品电子商务平台，打造农村市场营销网络。促进新民包印、辽中卡车及特种车、法库陶瓷、康平塑编等县域工业产业集群做大做强，不断增强县域经济发展实力。

　　(六)提升城乡建设管理水平

　　坚持高起点规划、高水平建设、高效能管理，不断提升城市的综合配套和服务功能，为加快建设国家中心城市奠定坚实基础。

　　加大城乡基础设施建设力度。加快城市快速路和轨道交通建设，启动南北二干线、地铁4号线、中央大街跨浑河桥等工程，加快建设地铁9号线和10号线，完成二环快速路二期工程，加强环路连接线建设。推进城际公路、交通枢纽和城市出口路建设，启动建设沈铁二号公路，加快南站主体工程和北站北广场建设步伐，完成迎宾路高架桥等节点工程。完善城市配套设施，实施浑河城市段南北两岸堤防工程，推进城市老旧泵站、积水点改造，实施燃气、供热、排水管网建设改造，加强地下空间开发利用。加快新城、新市镇基础设施建设，优化城市布局形态，积极稳妥推进新型城镇化。

　　提升城市管理精细化水平。加强信息技术应用和信息资源整合，加快智慧城市建设步伐。大力推进“公交都市”创建，强化公交场站、枢纽站、停车场建设，提升动、静态交通管理水平。加强城市进出口道路、城乡结合部、小街小巷、旧住宅区等薄弱区域环境综合整治。实施市政设施精细化维护管理。改造提升公园、广场、重点街路绿化景观。巩固全国文明城市创建工作成果，不断提高城市文明程度和市民文明素质。

　　(七)持续改善生态环境

　　生态环境关系人民幸福和城市可持续发展，必须下更大的决心、花更大的气力，加快建设生态宜居之都。

　　大力实施“蓝天”工程。抓好气化沈阳、城市抑尘、绿色交通、大气监控预警、拆小并大等七项工程。推进锅炉除尘和清洁能源改造，拆除50座锅炉房，推动华润热电等6家重污染企业搬迁。加大对秸秆焚烧的治理和管控力度，推进秸秆综合利用。

　　扎实推进“碧水”工程。实施西部污水处理厂二期、南运河截污工程，促进污水处理设施有效运行，提升城乡污水处理能力。加快康平卧龙湖国家湖泊环境保护试点工程建设，开展细河、满堂河等小流域治理，大力整治污水沟渠，努力改善城乡亲水环境。

　　强化环境综合整治。推进老虎冲和大辛垃圾焚烧及渗沥液处理等项目建设。全面开展宜居乡村建设。加大节能减排工作力度，推广使用新能源，积极发展循环经济，确保完成省下达的节能减排指标。实施棋盘山生态景观提升工程，大力开展植树造林。完成全市生态保护红线区划定工作，加快创建国家生态文明先行示范区。

　　(八)更加注重保障改善民生

　　人民群众对美好生活的向往就是我们的工作方向，要始终把保障和改善民生作为政府工作的出发点和落脚点，着力为人民群众做好事、办实事、解难事。

　　加大就业工作力度。完善就业创业扶持政策，多渠道开发就业岗位，突出抓好大学生等重点人群就业，做好实名制就业工作。强化以农民工为重点的特殊劳动群体权益保障，努力构建更加和谐的劳动关系。

　　完善社会保障体系。实施城乡居民社会养老保险并轨，启动机关事业单位养老保险制度改革。完善重特大疾病保障制度，全面启动居民大病医疗保险。继续加大社会保险扩面力度。提高企业退休人员养老金水平、城镇居民医保和新农合补助标准，进一步提升城乡居民最低生活保障标准和医疗救助水平。推进养老服务业综合改革试点，建设10个区域性居家养老服务中心，大力发展养老产业。加大扶贫帮困工作力度，大力发展慈善、残疾人事业。

　　改善城乡居民生活条件。完成保障性住房1万套，改造棚户区2.3万户、农村危房659户。实施50个旧住宅区节能暖房工程，改造提升200个旧住宅区居住环境，建立健全住宅区特别是旧住宅区长效管理机制。解决20.9万农村人口安全饮水问题。继续整修街巷路，维修改造500公里农村公路，新建150公里自然村连接道路，让城乡居民出行更便捷。

　　强化食品药品安全监管。加强农产品质量安全体系建设，推进食品药品重点环节、重点产品全程可追溯体系建设，严厉打击制假售假等不法行为。

　　(九)推动社会事业全面发展

　　把发展社会事业放在更加重要位置，加快推进公共服务均等化，不断提高人民群众的幸福指数。

　　优先发展教育事业。全面落实《沈阳市学前教育条例》，改造100所幼儿园，新增1万个普惠性幼儿园学位，启动中小学教学设备改造提升工程，全面完成义务教育和普通高中学校标准化建设，推进和平等5个区教育现代化建设，力争辽中、沈北等5个区县(市)通过国家义务教育发展基本均衡县验收。支持在沈高校发展，加快建设国家装备制造业职业教育试验区。

　　深化医药卫生体制改革。积极吸引社会资本参与公立医院改革发展，扩大优质医疗资源有效供给。推进卫生信息化建设，不断提升专科医院诊疗水平，加强基层医疗卫生机构建设，逐步建立科学的分级诊疗体系。推进健康服务业加快发展。

　　加快发展文化体育事业。加强现代公共文化服务体系建设，扎实推进文化基础设施提升工程，加快市少儿图书馆、青少年活动中心、妇女会馆、老年人大学等公共文化设施建设，创造生产更丰富、更优质的文化产品，大力发展文化产业。实施体育惠民工程，改造完善休闲健身公园，更新、安装户外健身器材，为推动全民健身提供更好的环境和条件。

　　努力提高社会治理水平。加强普法宣传教育，提升全民法治意识。加强信用体系建设，提高全社会信用水平。鼓励支持社会组织参与社会治理和公共服务。推进城乡社区服务体系建设，创新社区服务模式，全面实施和谐社区建设工程。加强防控体系建设，依法严厉打击各类违法犯罪行为。落实安全生产责任制，抓好道路交通、人员密集场所、建筑工地、危化品、特种装(设)备、地下管网和消防等各方面安全监管，坚决防范遏制重特大事故。加强社会矛盾化解工作，提高信访工作法治化水平，切实维护社会和谐稳定。

　　继续深化双拥共建。加强国防后备力量建设，扎实做好双拥模范城创建工作，推进军民融合深度发展。

　　(十)加强政府自身建设

　　加强政府自身建设，必须加快转变政府职能，改进工作作风，提升服务质量，不断增强政府的公信力和执行力。

　　深入推进依法行政。自觉接受市人大及其常委会的依法监督、市政协的民主监督和社会各方面监督，认真办理人大代表建议、议案和政协提案。完善重大行政决策合法性审查机制，落实风险评估、责任追究等制度。深化行政执法体制改革，建立完善重大行政处罚和行政强制决定审查备案制度，不断规范行政自由裁量权和行政执法检查权。坚持用法治思维谋划工作、用法治方式推动工作，全力打造职能科学、权责法定、执法严明、公开公正、廉洁高效的法治政府。

　　加快转变政府职能。深化政府机构改革，着力推进机构、职能、权限、程序、责任法定化，不断提升行政审批效能。加强市、区县(市)、乡镇三级服务体系建设，拓展和完善市长信箱、96123市民热线等民生诉求服务平台功能，进一步提高行政服务水平。全面推进政务公开，重点推进政府信息公开。健全政府预算体系，建立债务管理制度和风险预警处置机制。精心编制好“十三五”规划和各类专项规划，进一步提高规划的科学性和协调性。

　　切实改进工作作风。巩固和发展教育实践活动成果，认真落实“五个必须”要求，严守政治纪律和政治规矩，坚决克服畏难、观望、懈怠思想，坚决纠正不想为、不敢为、不作为，坚决抵制庸政、懒政、怠政。进一步教育引导全体政府工作人员，特别是各级领导干部自觉践行“三严三实”要求，强化责任意识和担当意识，恪守为民情怀，增强干事创业激情，不断提高政府工作质量和效率。

　　大力加强廉政建设。建立健全“五大系统”，不断增强制度的刚性和约束力。认真落实“一岗双责”，切实加强廉政教育,严格执行中央八项规定、《廉政准则》以及市委关于加强党风廉政建设的各项规定，严控“三公”经费支出，努力打造一支清正廉洁的公务员队伍。

　　各位代表，我们正处在变革与奋进的伟大时代。让我们更加紧密地团结在以习近平同志为总书记的党中央周围，在市委的正确领导下，在市人大及其常委会依法监督和市政协民主监督下，以更大的担当、更实的作风、更足的干劲，积极进取、攻坚克难，为加快实现沈阳全面振兴、把城市建设得更加美好、让城乡人民生活得更加幸福而不懈奋斗！
